# Association between serum magnesium levels and abdominal aorta calcification in patients with pre-dialysis chronic kidney disease stage 5

**DOI:** 10.1371/journal.pone.0253592

**Published:** 2021-06-18

**Authors:** Mayumi Ito, Makoto Yamaguchi, Takayuki Katsuno, Hironobu Nobata, Shiho Iwagaitsu, Hirokazu Sugiyama, Hiroshi Kinashi, Shogo Banno, Masahiko Ando, Yoko Kubo, Takuji Ishimoto, Yasuhiko Ito

**Affiliations:** 1 Department of Nephrology and Rheumatology, Aichi Medical University, Nagakute, Japan; 2 Data Coordinating Center, Department of Advanced Medicine, Nagoya University Hospital, Nagoya, Japan; 3 Department of Preventive Medicine, Nagoya University Graduate School of Medicine, Nagoya, Japan; 4 Department of Nephrology and Renal Replacement Therapy, Nagoya University Graduate School of Medicine, Nagoya, Japan; Nagoya University, JAPAN

## Abstract

**Background:**

Several studies have revealed the relationship between serum magnesium levels and vascular calcification in chronic kidney disease patients. Despite excellent predictability of abdominal aorta calcification for cardiovascular disease events, the relationship between serum magnesium levels and abdominal aorta calcification, as evaluated by quantitative methods, in pre-dialysis patients remains unclear. This study aimed to determine the abdominal aorta calcification volume using computerized tomography and its association with serum magnesium levels in pre-dialysis chronic kidney disease stage 5 patients.

**Methods:**

This single-center cross-sectional study included 100 consecutive patients with pre-dialysis chronic kidney disease stage 5 between January 2016 and May 2020 at Aichi Medical University Hospital, Japan. The relationships between serum magnesium levels and the abdominal aorta calcification volume were assessed using multiple linear regression models after adjusting for clinically relevant factors. We also assessed clinical factors that affect serum magnesium levels.

**Results:**

The mean serum magnesium level was 2.0 mg/dL (interquartile range, 1.8 to 2.3). Multivariate analyses revealed that a higher serum magnesium level (stand. β = -0.245, p = 0.010) was significantly associated with a reduced abdominal aorta calcification volume, and that a history of cardiovascular disease (stand. β = 0.3792, p < 0.001) and older age (stand. β = 0.278, p = 0.007) were significantly associated with an increased abdominal aorta calcification volume. Moreover, multivariate analysis showed that the use of proton pump inhibitor or potassium-competitive acid blocker was significantly associated with lower serum magnesium levels (stand. β = -0.246, p = 0.019).

**Conclusions:**

The present study revealed that the higher Mg level was significantly associated with lower volume of abdominal aorta calcification in pre-dialysis chronic kidney disease stage 5 patients. Further studies should be undertaken to determine the appropriate magnesium level to suppress vascular calcification.

## Introduction

Cardiovascular disease (CVD) is the most common cause of mortality in patients with chronic kidney disease (CKD) [1]. Although vascular calcification (VC) is considered an important risk factor for CVD [2], no specific therapy is available to prevent progression or to facilitate regression of VC; therefore, it is essential to identify clinical factors leading to VC and establish a preventive strategy for VC.

A large number of factors are responsible for development of VC in CKD, especially in mineral disorders [3], and phosphate (P) and calcium (Ca) are major contributors to development of medial calcification. In addition, several observational studies have shown that serum magnesium (Mg) levels inversely correlate with VC only in dialysis patients [4–7], and some experimental studies have demonstrated the importance of Mg as an anti-calcification mineral [8–15]. However, the serum Mg level is largely dependent on the dialysate Mg level [16]. Therefore, the findings of these observational studies that assessed dialysis patients should be interpreted cautiously; moreover, it remains unclear whether there is a similar relationship in pre-dialysis settings. Furthermore, methods for detection and quantification of VC, as well as baseline Mg levels, are different in various studies [7–15]. Thus, the relationship between the serum Mg level and VC remains to be investigated.

Accumulating evidence indicates that abdominal aorta calcification (AAC) is a good clinical target to evaluate atherosclerosis in the abdominal aorta and is a marker of subclinical atherosclerotic disease [17–24], and it correlates with asymptomatic coronary artery disease [17] and is highly predictive of subsequent VC outcomes in the general population [18, 19]. In CKD patients under hemodialysis and peritoneal dialysis, AAC could be used to predict CVD outcomes and total mortality [24]. AAC can be measured with methods, such as X-ray [25] or computerized tomography (CT) [26]. Compared with plain X-ray, CT can easily quantify AAC with objective and reproducible results [27, 28]; however, no studies have assessed the relationship between serum Mg levels and AAC evaluated using CT. Therefore, in the present study, we assessed the relationship between serum Mg levels and AAC using quantitative methods of VC with CT in patients with pre-dialysis CKD stage 5.

## Materials and methods

### Ethical statements

The study protocol was approved by the Ethics Committee of Aichi Medical University (approval number: 2018-H350; date: November 3, 2019). Due to the retrospective nature of the study, the need for patients’ informed consent was waived.

### Participants

**Fig 1:**
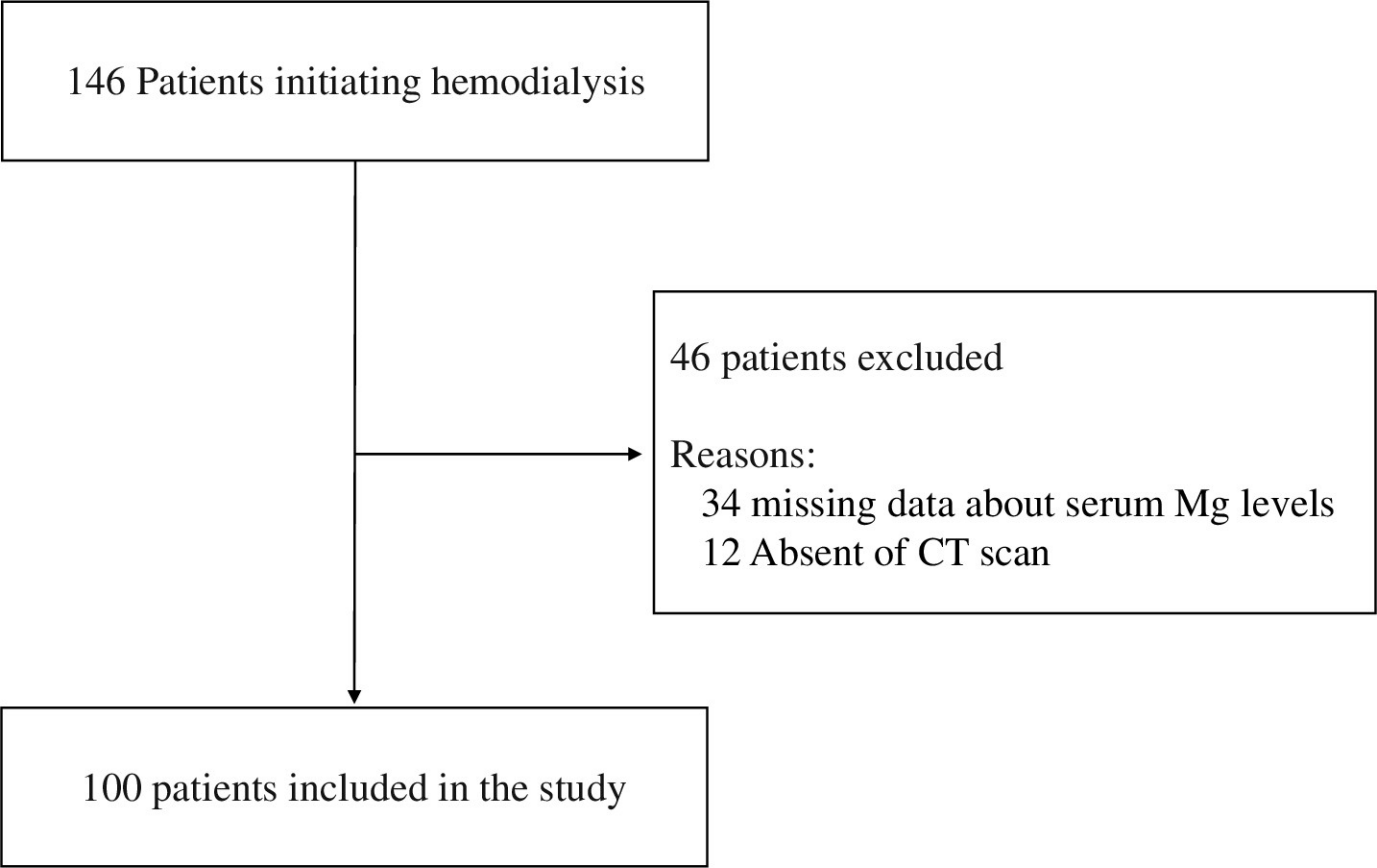
Flow diagram of patient selection.

The study included patients who were hospitalized for chronic hemodialysis between January 2016 and May 2020 at Aichi Medical University Hospital, Nagakute Aichi, Japan. Of the total 146 patients for whom hemodialysis was newly started, we included those who underwent at least two blood examinations of serum Ca, P, and Mg levels as well as abdominal CT scans that were used to screen for malignancy and vascular disease (aortic aneurysms, pleural effusion) by physician’s practice within 3 months prior to first hemodialysis. Finally, after excluding 34 patients with missing Mg data and 12 patients without abdominal CT scans, a total of 100 (68.5%) patients were included in the present study (Fig 1).

### Measurements

The following clinical data were obtained from medical records at the time of the first blood examination as baseline characteristics: age, sex, body mass index (BMI), and systolic/diastolic blood pressure (SBP/DBP); prescription of phosphate binders (calcium carbonate, lanthanum carbonate, sucroferric oxyhydroxide, and ferric citrate hydrate), proton pump inhibitors (PPI), potassium-competitive acid blocker (P-CAB), histamine-2 receptor blocker, statin, diuretic, antiplatelet drugs, vitamin K antagonist, antihypertensive medication, magnesium oxide (MgO), insulin, erythropoetine, and oral intravenous active vitamin D analog; and history of CVD (myocardial infarction, cerebral infarction, cerebral hemorrhage, and amputation of the extremities), diabetes mellitus (DM), hypercholesterolemia, hypertension (HT), and ever smoking. DM was defined as 1) a fasting serum glucose level of ≥ 126 mg/dL, 2) a random serum glucose level of ≥ 200 mg/dL, 3) a hemoglobin A1c level of ≥ 6.5%, and/or 4) prescription of glucose-lowering drugs, including insulin. Hypercholesterolemia was defined as a total serum cholesterol level of 240 mg/dL or the use of lipid-lowering medications. Hypertension was defined as taking antihypertensive medications or a systolic blood pressure (BP) of ≥140 mmHg and/or a diastolic BP of ≥90 mmHg. Smokers were defined as both current smokers and ex-smokers.

Laboratory measurements (serum levels of albumin, Ca, P, Mg, creatinine, estimate glomerular filtration rate (eGFR), total cholesterol, high and low-density lipoprotein (HDL and LDL) cholesterol, triglyceride (TG), hemoglobin, C-reactive protein (CRP), alkaline phosphatase, and intact parathyroid hormone [iPTH]) were averaged using the laboratory values collected during the three months prior to the first dialysis. If the serum albumin level was < 4.0 g/dL, the serum Ca level was adjusted as follows: corrected serum Ca level (mg/dL) = measured serum Ca level (mg/dL) + (4.0—serum albumin level (g/dL).

### Evaluation of abdominal aorta calcification

AAC volume was evaluated using CT scans. Abdominal images were obtained using multi-detector CT scanners at the Aich Medical University of Nagakute, Aichi (SOMATOM Definition AS+ 128 slices, 64-row [Siemens] and SOMATOM Definition Flash [Siemens]). Images were reconstructed with a slice thickness of 2 mm (multi-detector scanners). For equal comparisons among participants with different abdominal aortic lengths, an 8 cm segment proximal to the aortic bifurcation was used to quantify AAC with the method of Forbang NI et al. [29].

The volume of AAC (cm^3^) was quantified using the definition of calcification in the Agatston score [17]. CT values of Hounsfield Units greater than 130 in each slice were defined as dominant calcification, and calcification of the abdominal aortic wall was identified in each slice of 2.5 mm thickness; they were automatically calculated by image processing software (Ziostation2, Ziosoft, Inc.).

### Statistical analyses

We stratified patients into the following 3 categories according to the tertile of serum Mg levels: lower (< 1.9 mg/dL), intermediate (1.9–2.2 mg/dL), and higher (≥ 2.2 mg/dL). Clinical characteristics were compared using the Kruskal–Wallis test followed by Mann–Whitney post-hoc analysis with Bonferroni correction or Pearson’s chi-square test, when appropriate. To determine the factors that were independently associated with VC, we evaluated the AAC volume using univariate linear regression analysis. Multiple linear regression analysis for the AAC volume was performed with adjustment for the following clinically relevant factors: age; sex; SBP; serum adj. Ca, Mg, P, and iPTH level; and history of CVD, DM, and HT. Correlations between continuous data were assessed using Pearson’s or Spearman’s test, as appropriate. Furthermore, to determine the clinical factors associated with the serum Mg level, multiple linear regression analysis was performed with adjustment for the following clinically relevant factors: age; sex; serum albumin and iPTH level; prescription of phosphate binders and MgO; and history of CVD and DM. The level of statistical significance was set at P < 0.05. All analyses were performed using SAS version 9.4 (SAS Institute, Inc, Cary, NC) and SPSS statistics version 26 (Stata Corp, College Station, USA).

## Results

### Baseline characteristics

Baseline characteristics of patients according to the tertiles of serum Mg levels are outlined in Table 1. Among all patients, 31% were in the lower Mg group (< 1.9 mg/dL), 38% were in the intermediate Mg group (1.9–2.2 mg/dL), and 31% were in the higher Mg group (≥ 2.2 mg/dL). Patients in the higher Mg group showed higher adjusted serum Ca level (p = 0.023), lower AAC volume (p < 0.001), lower prevalence of PPI/P-CAB use (p = 0.020), and higher prevalence of MgO use (p = 0.032) than those in the lower Mg group. Other findings were comparable among the three groups.

**Table 1:** Baseline characteristics of patients stratified by Mg categories.

Characteristics		Magnesium groups (range of serum magnesium levels [mg/dL])			P values
	Total	lower (< 1.9)	intermediate (≥ 1.9, < 2.2)	higher (≥ 2.2)	
	n = 100	n = 31	n = 38	n = 31	
Age, year	72 [63, 79]	73 [64, 80]	75 [64, 81]	71 [55, 76]	.352
Male, n (%)	69 (69)	21 (67)	24 (63)	24 (77)	.437
BMI, kg/m^2^	21 [19, 24]	22 [15, 27]	20 [13, 37]	21 [14, 34]	.444
History of CVD, n (%)	28 (28.0)	8 (25.8)	13 (34.2)	7 (22.6)	.535
smoker, n (%)	55 (55.0)	17 (54.8)	21 (55.3)	17 (56.7)	.989
DM, n (%)	59 (59.0)	21 (67.7)	20 (52.6)	18 (58.1)	.443
Duration of DM, year	9 [0, 21]	8 [0, 23.5]	0 [0, 20.75]	18 [0, 20]	.434
HT, n (%)	95 (95.0)	31 (100.0)	35 (92.1)	29 (93.5)	.295
Hypercholesterolemia, n (%)	38 (38.8)	12 (40.0)	11 (29.7)	15 (48.4)	.286
SBP, mmHg	150 [131, 164]	146 [132, 162]	154 [132, 165]	150 [134, 162]	.977
DPB, mmHg	75 [65, 84]	75 [64, 83]	76 [64, 83]	74 [69, 84]	.969
eGFR, mL/min/1.73m^2^	5 [4.6, 7]	6 [4, 7]	5 [5, 6.8]	6 [4.3, 7.5]	.746
P, mg/dL	5.4 [4.5, 6.7]	5.7 [4.7, 6.6]	5.5 [4.8, 6.6]	5.1 [4.5, 6.3]	.661
Adj. Ca, mg/dL	9.0 [8.6, 9.3]	8.8 [8.5, 9.1]	9.1 [8.9, 9.3]	9.0 [8.5, 9.3]	.023
Ca×P	49.5 [43.4, 62.5]	49.5 [44.1, 62.6]	50.9 [43.7, 61.7]	48.0 [41.4, 56.1]	.437
CRP, mg/dL	0.41 [0.16, 2.01]	0.32 [0.14, 2.39]	0.44 [0.18, 2.02]	0.30 [0.10, 0.85]	.666
Hb, g/dL	9.9 [8.8, 10.9]	9.6 [8.8, 10.4]	10.3 [8.8, 11.2]	9.8 [8.7, 10.7]	.439
Alb,g/dL	3.1 [2.7, 3.5]	3.1 [2.7, 3.5]	3.1 [2.6, 3.5]	3.1 [2.8, 3.6]	.652
ALP, IU	234 [179, 301]	223 [187, 280]	245 [181, 332]	233 [177, 296]	.800
iPTH, pg/mL	251 [154, 381]	238 [189, 369]	213 [126, 351]	264 [192, 387]	.634
TC, mg/dL	171 [146, 199]	163 [136, 180]	171 [141, 206]	176 [165, 198]	.231
HDL-C, mg/dL	45 [35.5, 57]	48 [38.5, 66]	46 [35, 51]	43 [36.5, 54.5]	.299
LDL-C, mg/dL	92 [72, 111]	86 [70, 106]	95 [70, 132]	94 [78, 107]	.566
Hyper-LDL-cholesterolemia (LDL-C ≥140 mg/dL), n (%)	11 (12.0)	3 (10.7)	4 (11.1)	4 (14.3)	.900
TG, mg/dL	113 [84, 153]	118 [83, 147]	102 [79, 143]	113 [98, 157]	.488
Cr, mg/dL	7.54 [6.23, 9.73]	8.10 [7.03, 9.83]	7.15 [5.92, 8.15]	7.88 [6.19, 10.51]	.288
Fe, μg/dL	51 [36, 69]	51 [36, 54]	52 [39, 71]	55 [36, 69]	.509
UPCR, g/g∙Cr	4.77[2.17, 7.45]	6.33 [3.52, 8.99]	3.96 [2.48, 7.02]	4.16 [1.83, 6.08]	.076
Prescription					
Phosphate binder	36 (36.0)	10 (32.3)	15 (39.5)	11 (35.5)	.822
Calcium carbonate, n (%)	19 (19)	7 (22.6)	3 (7.9)	9 (29.0)	0.07
Lanthanum, n (%)	15 (15)	5 (16.1)	4 (7.9)	7 (22.6)	.231
Sucroferric oxyhydroxide, n (%)	1 (1)	0 (0)	0 (0)	1 (3.2)	.325
Ferric Citrate Hydrate, n (%)	9 (9)	4 (12.9)	5 (13.2)	0 (0)	.108
Duration of Phosphate binder,(month)	3 [2, 5.5]	4 [2, 6]	2 [2, 5.5]	4 [2, 4.5]	.781
MgO, n (%)	3 (3)	0 (0)	0 (0)	3 (9.7)	.032
Duration of MgO, (month)	2 [2, 10]	0	0	2 [2, 10]	.033
PPI/P-CAB, n (%)	31(31)	14 (45.2)	13 (34.2)	4(12.9)	.020
Duration of PPI/P-CAB, (year)	2 [1, 4]	3 [2, 5]	2 [1, 3.5]	2 [1.5, 3]	.554
*Vitamin D therapy (p.o.), n (%)	37 (37)	13 (41.9)	14 (36.8)	10 (32.3)	.732
H_2_ blocker, n (%)	1(1)	0 (0)	1 (2.6)	0 (0)	.439
Warfarin, n (%)	8(8)	1 (4)	4 (12.5)	3 (10.3)	.533
Anti-platelet drug, n (%)	16(16)	7 (22.6)	7 (18.4)	2 (6.5)	.195
Diuretic, n(%)	70(70)	21 (67.7)	30 (78.9)	19 (61.3)	.267
Statin, n(%)	32(32)	14 (45.2)	9 (23.7)	9 (31.0)	.163
ESA, n (%)	75 (75)	21 (67.7)	29 (76.3)	25 (80.6)	.488
Volume of AAC, cm ^3^	3.25 [1.62, 7.10]	4.43 [2.98, 7.46]	3.80 [2.01, 8.58]	1.84 [0.47, 4.15]	< .001
Relative Volume of AAC, (cm ^3^/ m^2^ BSA)	2.24 [0.96, 4.60]	3.55 [1.86, 4.78]	2.59 [1.26, 5.60]	1.15 [0.22, 2.49]	.001

*25OH vitamin D, alfacalcidol or eldecalcitol.

Data are presented as number (percent) or median [interquartile range].

Abbreviations: BMI, body mass index; CVD, cardiovascular disease; DM, diabetes mellitus; HT, hypertension; SBP, Systolic blood pressure; DBP, diastolic blood pressure; eGFR, estimate glomerular filtration rate; P, phosphate; adj.Ca, adjusted calcium; CRP, C-reactive protein; Hb, hemoglobin; Alb, albumin; ALP, alkaline phosphatase; iPTH, intact parathyroid hormone; TC, total cholesterol; HDL, high density lipoprotein; LDL, low-density lipoprotein; TG, triglyceride; Cr, creatinine; Fe, ferritin; UPCR, urine protein to creatinine ratio; MgO, magnesium oxide; PPI, proton pump inhibitor; P-CAB, potassium-competitive acid blocker; H_2_, histamine-2 receptor; ESA, erythropoiesis stimulating agents; AAC, abdominal aortic calcification; BSA, body surface area.

### Clinical factors affecting the AAC volume

In the univariate analysis, older age, higher serum adjusted Ca levels, and a history of CVD were significantly associated with an increased AAC volume, and higher serum Mg levels and the use of MgO were significantly associated with a reduced AAC volume (p < 0.005) (Table 2). Multiple linear regression analysis revealed that a history of CVD (stand. β = 0.379, p < 0.001) and older age (stand. β = 0.278, p = 0.007) were significantly associated with an increased AAC volume, and higher Mg levels (stand. β = -0.245, p = 0.010) were significantly associated with a reduced AAC volume (Table 3). As well as in the relative AAC volume by BSA adjustment, these results had not been changed significantly (S1 Table).

**Table 2:** Univariate linear regression for AAC volume.

Variable	stand. B	SE	T	P values
Age	.354	.028	3.759	< .001
Male	-.075	.862	-.743	.459
BMI	-.081	.094	-.803	.424
History of CVD	.392	.819	4.221	< .001
Smoker	.038	.810	.379	.705
DM	.103	.808	1.025	.308
Duration of DM	.152	.030	1.527	.130
HT	.188	1.801	1.899	.061
Hypercholesterolemia	.109	.830	1.074	.286
SBP, mmHg	-.076	.014	-.755	.452
P	-.081	.272	-.807	.422
Adj. Ca	.213	.524	2.155	.034
Ca×P	-.002	.026	-.023	.982
iPTH	-.001	.001	-.645	.521
Magnesium	-.254	.958	-2.600	.011
Albumin	.029	.682	.290	.773
TC	.099	.010	.973	.333
HDL-C	.012	.026	.112	.911
LDL-C	.084	.012	.796	.428
TG	.122	.004	1.177	.242
CRP	-.109	.139	-1.081	.282
Prescription				
Phosphate binder	.055	.831	.548	.585
Duration of Phosphate binder	-.163	.087	-.952	.348
PPI/P-CAB	.028	.831	.548	.585
Duration of PPI/P-CAB	.025	.276	.244	.807
MgO	-.720	1.437	2.236	.028
Duration of MgO	-.876	.190	-1.820	.320
H_2_ blocker	.060	.849	.273	.785
Warfarin	.220	4.009	.593	.555
Statin	.338	2.341	-.308	.759
Insulin	-.110	.993	-1.091	.278
ESA	.024	.923	.239	.812

Abbreviations: stand. β, standardized β-coefficient; SE, standard error of stand. β; BMI, body mass index; CVD, cardiovascular disease; DM, diabetes mellitus; HT, hypertension; SBP, Systolic blood pressure; eGFR, estimate glomerular filtration rate; P, phosphate; adj.Ca, adjusted calcium; TC, total cholesterol; HDL, high density lipoprotein; LDL, low-density lipoprotein; TG, triglyceride; iPTH, intact parathyroid hormone; CRP, C-reactive protein; P-CAB, potassium-competitive acid blocker; H_2_, histamine-2 receptor; MgO, magnesium oxide; PPI, proton pump inhibitor; AAC, abdominal aortic calcification; ESA, erythropoiesis stimulating agents.

**Table 3:** Multiple linear regression for AAC volume.

Variable	stand. β	SE	T	P value
Age	.278	.029	2.747	.007
Male	-.050	.893	-.472	.638
History of CVD	.379	.857	3.941	< .001
DM	.154	.754	1.609	.112
HT	.093	1.714	.913	.364
SBP	-.045	.015	-.450	.654
P	.090	.257	.898	.372
adj. Ca	.062	.556	.584	.561
iPTH	-.103	.001	-1.018	.312
Magnesium	-.245	.877	-2.658	.010

Abbreviations: stand. β, standardized β-coefficient; SE, standard error of stand. β; CVD, cardiovascular disease; DM, diabetes mellitus; HT, hypertension; SBP, Systolic blood pressure; P, phosphate; adj.Ca, adjusted calcium; iPTH, intact parathyroid hormone; AAC, abdominal aortic calcification.

### Clinical factors affecting serum Mg levels

Univariate analysis showed that the use of PPI/P-CAB was associated with lower Mg levels, and the use of MgO was associated with higher Mg levels (p < 0.005) (S1 Table). Multivariate analysis showed that the use of PPI/P-CAB (β = -0.246, p = 0.019) was associated with lower serum Mg levels, and the use of MgO was associated with higher serum Mg levels (β = 0.295, p = 0.005) (Table 4).

**Table 4:** Multiple linear regression for serum Mg levels.

Variable	stand. β	SE	T	P value
Age	.032	.003	.306	.760
Male	.133	.097	1.218	.227
History of CVD	.037	.098	.356	.723
DM	-.071	.084	-.692	.491
Albumin	.003	.071	.031	.975
iPTH	-.059	< .001	-.561	.576
Prescription				
PPI/P-CAB	-.246	.096	-2.387	.019
MgO	.295	.233	2.870	.005

Abbreviations: stand. β, standardized β-coefficient; SE, standard error of stand. β; BMI, body mass index; CVD, cardiovascular disease; DM, diabetes mellitus; iPTH, intact parathyroid hormone; PPI, proton pump inhibitor; P-CAB, potassium-competitive acid blocker; MgO, magnesium oxide.

## Discussion

The present study revealed that higher Mg levels were significantly associated with lower volume of abdominal aorta calcification in pre-dialysis chronic kidney disease stage 5 patients. This finding suggests that the higher serum Mg level might suppress VC in the abdominal aorta. To the best of our knowledge, this study is the first to evaluate the relationship between the serum Mg level and AAC calculated by quantitative methods with CT in pre-dialysis CKD stage 5 patients.

Previous studies have demonstrated that serum Mg levels inversely correlate with VC in dialysis patients [4–7]. For example, one study including 52 hemodialysis patients with VC and 338 without VC found that serum Mg levels were independently associated with VC after adjusting for age, sex, hemodialysis vintage, Ca, P, and PTH concentration (odds ratio = 0.28, 95% CI = 0.09–0.92) [5]; however, that study focused on only non-DM patients, other arteries (except for hand arteries) were not evaluated for VC, and the methods for calculating VC were not quantitative. In addition, another study, including 80 peritoneal dialysis patients with DM or non-DM, evaluated VC using XP in the abdominal aorta and found that a higher serum Mg level was associated with decreased AAC after adjusting for age, serum P, PTH, LDL-C, smoking history, and diabetes [6]; the serum Mg level was 0.84 mmol/L, which is almost the same as that in our cohort but is lower than that in the hemodialysis population [30–33]. The results of serum Mg concentration in that study [6] might be significantly influenced by low Mg-contained peritoneal dialysate solution.

With regard to clinical factors other than Mg associated with AAC, studies have shown that high levels of serum P, Ca, and iPTH are risk factors for VC [34]. IPTH can increase the expression of the cartilage matrix and enhance the intracellular Ca levels, thereby promoting the formation of VC [35, 36]. Furthermore, a recent RCT reported that strict phosphate control delays the progression of coronary artery calcification in a dialysis population [37]. However, our study showed that serum levels of Ca, P, and iPTH were not associated with AAC. There are several possible reasons for this, such as study design, time of testing, and number of times tested. We postulate that this difference is probably because the serum P, Ca, and iPTH levels in most of our patients were within the target range and our small sample size, leading to a low statistical power which is unable to estimate the association between P, Ca, and iPTH with AAC. Additionally, serum P level might be influenced by a patient’s nutritional state, but the amount of phosphate in the diet could not be evaluated in our study. For example, in patients who had had appetite loss due to uremia, phosphate level at the time of starting dialysis might be lower, resulting in a possible underestimation of the influence of serum P levels on AAC. Therefore, more well-designed RCTs should be undertaken in order to clarify whether strict phosphate control delays progression of AAC in pre-dialysis CKD patients. Furthermore, as for the phosphate level control, although phosphate binders have been demonstrated to improve mineral metabolism and reduce cardiovascular mortality and morbidity in end-stage chronic kidney disease [38], in this study, phosphate binders were not associated with AAC and serum Mg levels. This may be due to the retrospective nature of the present study; the types and amount of phosphate binders were not standardized but rather left to each physician’s discretion; and the short duration of prescription and therefore had a clinically small impact on anti-vascular calcification. Therefore, in our study, the effect of phosphate binders on phosphate and Mg levels could not be precisely evaluated. In order to clarify the possible effect of phosphate binders on AAC, a well-designed RCT should be performed in the future.

Regarding other factors that may be responsible for development of VC, decreased eGFR levels have been reported to be related to an increased AAC [39]; however our results did not show any significant association between eGFR level and AAC. This is possibly due to the fact that our study only included patients with CKD stage 5, just before starting dialysis with eGFR levels within a narrow range, thus, we postulate that these small differences in eGFR levels have a negligible influence on AAC. Further studies including patients with various eGFR level should be undertaken in order to reveal the relationship between eGFR level and AAC in the future.

In addition, some previous reports showed that erythropoietin (EPO) and hypoxia-inducible factor (HIF) have been associated with vascular calcification. One study reported that EPO attenuates vascular calcification by inhibiting endoplasmic reticulum stress in rats with CKD [40]; meanwhile, other studies have reported that EPO and HIF-1 can promote vascular calcification in CKD, suggesting that inappropriate use of EPO and prolyl HIF-hydroxylase (PH) enzyme (HIF-PH) inhibitors in CKD patients might aggravate vascular calcification and elicit adverse clinical consequences [41, 42]. In this study, no significant association between use of ESA and AAC was found. However, in order to clarify the influence of ESA and HIF-PH for anti-vascular calcification, large scale, well designed RCTs should be undertaken in the future.

As for clinical factors that affect serum Mg levels, studies have shown that some medications may affect serum Mg levels in CKD patients. Consistently, the present study revealed that the use of PPI/P-CAB was a risk factor for lower serum Mg levels, probably because anti-gastric acid medication hampers intestinal Mg reabsorption and consequently increases the risk of hypomagnesemia [43, 44].

A number of experimental studies have suggested that administration of Mg prevents VC [45–47], and the potential mechanisms include the inhibition of calcium-phosphate crystal growth in the circulation, thus decreasing calcium-phosphate deposition, and prevention of the phenotype change of the vascular smooth muscle to osteoblasts [45, 46]. However, no large-scale clinical studies have elucidated the clinical efficacy of Mg supplements in CKD. Therefore, well-designed randomized controlled trials are needed to illustrate the preventive effects of Mg on VC in CKD patients.

There are some limitations to this study. First, the study was a cross-sectional study and had a small sample size; therefore, the causal relationship between Mg and VC could not be fully determined, and there might be other confounding factors that could not be adjusted. Second, we could not obtain precise information regarding changes in dosage and serum levels for MgO, PPI/P-CAB, and phosphate binders over the study period, and the prescription methods were not standardized. Furthermore, we only assessed the relationship between Mg levels and AAC just before starting dialysis; therefore, it is difficult to conclude that there is a close relationship between Mg levels just before dialysis and AAC. Further studies should obtain the data about these drug prescriptions and Mg levels over the entire period of ongoing vascular calcification. Third, we did not evaluate the AAC progression and CVD prognosis; therefore, well-designed longitudinal studies should be undertaken to assess these points in the future.

Fourth, we did not assess the difference between medial and intimal calcification, although intimal calcification has been demonstrated to be associated with worse survival compared with medial calcification [47, 48]. Therefore, further studies are needed to address these limitations.

In conclusion, this cohort study found that higher Mg levels were associated with lower volume of AAC in pre-dialysis chronic kidney disease stage 5 patients. Further studies should be undertaken to identify the appropriate Mg level for suppressing VC in CKD patients.

## Supporting information

S1 Table Multiple linear regression for relative AAC volume (cm ^3^/ m^2^ BSA). (DOCX)

S2 Table Univariate linear regression for serum Mg levels. (DOCX)
