# Supplementary material for: Association between serum magnesium levels and abdominal aorta calcification in patients with pre-dialysis chronic kidney disease stage 5
Source: PLoS One. 2021 Jun 18;16(6):e0253592. doi: 10.1371/journal.pone.0253592 (PMC8213142; doi:10.1371/journal.pone.0253592)
Supplement: S1 Table — (DOCX) [file pone.0253592.s001.docx]

**S1 Table. Multiple linear regression for relative AAC volume (cm ³/ m² BSA)**

| **Variable** | **stand. β** | **SE** | **T** | **P values** |
| --- | --- | --- | --- | --- |
| Age | .317 | .018 | 3.186 | .002 |
| Male | -.150 | .563 | -1.450 | .151 |
| history of CVD | .358 | .540 | 3.781 | <.001 |
| DM | .151 | .476 | 1.607 | .112 |
| HT | .102 | 1.081 | 1.023 | .309 |
| SBP | -.012 | .009 | -.120 | .905 |
| phosphate | .071 | .162 | .719 | .474 |
| adj. Ca | .038 | .351 | .361 | .719 |
| iPTH | -.115 | .001 | -1.155 | .251 |
| magnesium | -.241 | .553 | -2.659 | .010 |

Abbreviations: stand. β, standardized β-coefficient; SE, standard error of stand. β; BMI, body mass index; CVD, cardiovascular disease; P, phosphate; adj.Ca, adjusted calcium; iPTH, intact parathyroid hormone; UPCR, urine protein to creatinine ratio; PPI, proton pump inhibitor; P-CAB, potassium-competitive acid blocker; H_2_, histamine-2 receptor; MgO, magnesium oxide; AAC, abdominal aortic calcification.
