# Supplementary material for: Association between serum magnesium levels and abdominal aorta calcification in patients with pre-dialysis chronic kidney disease stage 5
Source: PLoS One. 2021 Jun 18;16(6):e0253592. doi: 10.1371/journal.pone.0253592 (PMC8213142; doi:10.1371/journal.pone.0253592)
Supplement: S2 Table — (DOCX) [file pone.0253592.s002.docx]

**S2 Table. Univariate linear regression for serum Mg levels.**

| **Variable** | **stand. β** | **SE** | **T** | **P values** |
| --- | --- | --- | --- | --- |
| Age | .008 | .003 | .075 | .941 |
| Male | .090 | .088 | .899 | .371 |
| BMI | -.042 | .010 | -.412 | .681 |
| History of CVD | .006 | .091 | .059 | .953 |
| P | -.027 | .028 | -.272 | .786 |
| Adj. Ca | .036 | .055 | .359 | .720 |
| Albumin | .010 | .070 | .096 | .923 |
| iPTH | -.062 | .000 | -.575 | .567 |
| UPCR | -.096 | .002 | -.925 | .357 |
| Prescription |  | | | |
| Phosphate binder | .154 | .084 | 1.546 | .125 |
| Duration of　Phosphate binder | -.060 | .009 | -.345 | .732 |
| PPI/P-CAB | -.322 | .082 | -3.373 | .001 |
| Duration of　PPI/P-CAB | -.095 | .030 | -.515 | .610 |
|  |  |  |  |  |
| MgO | .329 | .226 | 3.445 | .001 |
| Duration of　MgO | -.477 | .054 | -.543 | .683 |
| H_2_ blocker | -.047 | .409 | -.466 | .642 |
| Warfarin | .037 | .150 | .366 | .715 |
| Diuretic | -.084 | .089 | -.834 | .406 |
| Statin | -.062 | .085 | -.610 | .543 |

Abbreviations: stand. β, standardized β-coefficient; SE, standard error of stand. β; BMI, body mass index; CVD, cardiovascular disease; P, phosphate; adj.Ca, adjusted calcium; iPTH, intact parathyroid hormone; UPCR, urine protein to creatinine ratio; PPI, proton pump inhibitor; P-CAB, potassium-competitive acid blocker; H_2_, histamine-2 receptor; MgO, magnesium oxide; AAC, abdominal aortic calcification.
